# Effect of Simultaneous Exposure of Pigs to *Streptococcus suis* Serotypes 2 and 9 on Their Colonization and Transmission, and on Mortality

**DOI:** 10.3390/pathogens6040046

**Published:** 2017-09-27

**Authors:** Niels Dekker, Annemarie Bouma, Ineke Daemen, Hans Vernooij, Leo van Leengoed, Jaap A. Wagenaar, Arjan Stegeman

**Affiliations:** 1 Department of Farm Animal Health, Faculty of Veterinary Medicine, Utrecht University, 3584 CL Utrecht, The Netherlands; A.Bouma@minez.nl (A.B.); A.J.J.M.Daemen@uu.nl (I.D.); J.C.M.Vernooij@uu.nl (H.V.); L.vanLeengoed@uu.nl (L.v.L.); J.A.Stegeman@uu.nl (A.S.); 2 Department of Infectious Diseases and Immunology, Faculty of Veterinary Medicine, Utrecht University, 3584 CL Utrecht, The Netherlands; J.Wagenaar@uu.nl

**Keywords:** *Streptococcus suis*, serotype 2, serotype 9, pig, transmission, colonization, tonsil, nose, survival, qPCR

## Abstract

The distribution of *Streptococcus suis* serotypes isolated from clinically infected pigs differs between geographical areas, and varies over time. In several European countries, predomination of serotype 2 has changed to serotype 9. We hypothesize a relation, with one serotype affecting the other in colonization and invasion. The aim of this study was to evaluate whether simultaneous exposure of pigs to serotypes 2 and 9 affects colonization and transmission of each type, and mortality. Thirty-six caesarean-derived/colostrum-deprived piglets were randomly assigned to three groups, and there housed pair-wise. At six weeks old, one pig per pair was inoculated with either one (serotype 2 or 9; mono-group) or two serotypes simultaneously (dual-group); the other pig was contact-exposed. Tonsillar and nasal samples were collected within three weeks post inoculation. Bacterial loads in samples were quantified using multiplex real-time polymerase chain reaction (PCR). Transmission rates of the serotypes among pigs were estimated using a mathematical Susceptible-Infectious (SI) model. Bacterial loads and transmission rates did not differ significantly between serotypes. Compared to the mono-group, in the dual-group the average serotype 2 load in tonsillar samples from contact pigs was reduced on days 1 to 4 and on day 6. Simultaneous exposure to the serotypes reduced the mortality hazard 6.3 times (95% C.I.: 2.0–19.8) compared to exposure to serotype 2 only, and increased it 6.6 times (95% C.I.: 1.4–30.9) compared to exposure to serotype 9 only. This study indicates that serotype 2 load and mortality were affected in pigs exposed to these two serotypes.

## 1. Introduction

*Streptococcus suis* (*S. suis*) is a pathogen for both pigs and humans, that is endemically present in commercial pig herds worldwide [1], and seems to be emerging in humans [2]. The natural habitat of *S. suis* in pigs is the upper respiratory tract (especially the tonsils and nasal cavities), as well as the gastrointestinal and genital tracts [1,3,4,5]. The respiratory route is generally thought to be the main route of horizontal transmission among pigs, and especially the tonsils are considered important reservoirs of *S. suis* [1,5]. According to the most accepted hypothesis, *S. suis* breaches the mucosal epithelium at either the upper respiratory tract or the gastrointestinal tract, and reaches the bloodstream, leading to systemic infection [3,4,5]. Although pigs of all ages may be colonized, severe systemic infections, characterised by meningitis, arthritis, endocarditis, pneumonia, and septicaemia, seem to occur mainly in young pigs (5–10 weeks of age) [1,3,5]. These clinical outbreaks result in considerable animal welfare problems and economic losses. 

Based on differences in capsular polysaccharide (CPS) antigens, several serotypes of *S. suis* can be distinguished. So far, 33 serotypes of *S. suis* are known, although some are under debate as to whether they belong to the *S. suis* taxon, and new *cps* loci have been described in previously untypeable strains [6,7,8]. Often a pig is colonized by more than one serotype [9,10], but usually only one serotype is isolated from the internal organs of a pig that has suffered from a severe systemic infection [1]. The distribution of serotypes among clinical isolates differs between regions, and may also vary over time. This has been shown in many studies carried out in several regions/countries [6,11]. Results from some studies (performed in e.g., United Kingdom, Denmark, Spain, and the Netherlands) indicated that the predominant serotypes isolated from clinical cases have changed over the last decades [12,13,14]. In the Netherlands, for example, serotype 2 was found most often amongst isolates for several years (32% serotype 2 versus 68% of a variety of serotypes), but from 1996 onwards, serotype 9 has been isolated more frequently (56–58% type 9 versus 23–28% type 2) [15,16,17]. In Spain an even stronger increase in serotype 9 contribution was observed, i.e., from 4.4% or lower in the period 1991–1995 [16,18,19], to 54–65% in the period 1998–2002 [6,14,20]. To develop sustainable control measures effective in reducing the incidence of *S. suis* infections, insight in the mechanism(s) underlying this change in serotype distribution among clinical isolates is needed.

The probability that a pig develops a clinical infection due to a certain serotype depends on the efficiency of that type to colonize the mucosa, its virulence or invasive capacity, and by the susceptibility of the pig. These characteristics may be affected by the presence of other serotypes of the same bacterial species, as has been shown for *Streptococcus pneumoniae* (*S. pneumoniae*) in humans [21,22,23,24], and in mouse models [25,26,27,28]. On farm level, this ‘exposure history’ depends on the relative rates at which various serotypes can spread among pigs.

The effect of exposure of pigs to more than one serotype on colonization of each of these and on the clinical outcome of an infection has been studied experimentally using pigs originating from a conventional farm [29]. In that study the presence of serotypes 2 and 9 in piglets from dams positively tested for both types was determined at the end of the lactation period at four weeks of age. However, as timing of exposure to either of the two serotypes was unknown in this study, its data cannot be used to quantify the relative transmission rates of serotypes amongst pigs.

Therefore, we carried out transmission experiments to study the transmission rates of serotypes 2 and 9 among piglets. The objective of this study was to evaluate whether *S. suis* serotypes 2 and 9, when inoculated simultaneously, would affect their respective capacity to colonize mucosa and to spread among pigs, and whether the clinical outcome of a systemic infection caused by one serotype was altered by the presence of the other. Results may help explain why a serotype shift from 2 to 9 has occurred.

## 2. Results

### 2.1. Colonization

Before inoculation all pigs tested *S. suis* negative by selective bacterial examination (SBE) and by multiplex real-time quantitative PCR (qPCR), within their limits of detection. All inoculated pigs tested positive at the first day after inoculation. All contact pigs became *S. suis* positive within two days post-exposure (DPE) to *S. suis* inoculated pigs. All positive pigs remained positive during the observational period. 

**Figure 1:**
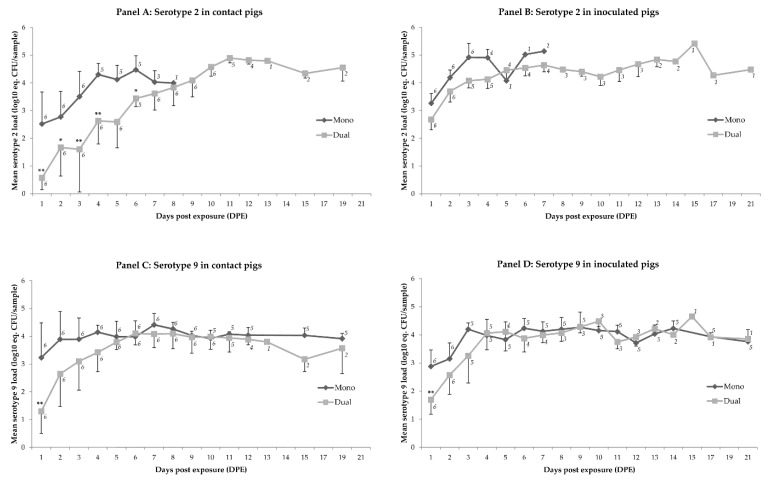
Mean loads of *S. suis* serotype 2 (panels A and B) or serotype 9 (panels C and D) in tonsillar samples (log_10_ eq. CFU/sample) per day post exposure (DPE) visualized for animal categories that differ in type of exposure (i.e., mono or dual), and in mode of infection (i.e., contact or inoculated). Significant differences in levels between mono- and dual-exposed pigs with the same infection mode are marked with (*) if *p* < 0.05 or (**) if *p* < 0.01. The bar at each data point reflects the standard deviation. Number of observations per data point is shown close to that point.

In tonsillar samples, no significant differences in loads were observed at any of the sampling days when exposure to serotype 2 only was compared to exposure to serotype 9 only (*p* = 0.99). Tonsillar serotype 2 loads of inoculated pigs did not differ between the pigs exposed to one serotype (referred to as ‘mono-group’) and two serotypes (referred to as ‘dual-group’); the mean loads in contact pigs in the dual-group were between 1.4 and 1.8 equivalent log_10_ colony forming units per sample (log_10_ eq. CFU/sample) reduced on all first four days, and on day 6 after exposure. No differences were observed in tonsillar serotype 9 loads in the mono-group compared to the dual-group, except for day 1 after exposure. At that moment the dual-group showed a 1.6 and 1.7 log_10_ eq. CFU/sample reduction in inoculated and contact pigs, respectively (Figure 1; Table 1 and Table 2).

Tonsillar serotype 2 loads in inoculated pigs of the mono-group did not differ from the loads in contact pigs, except for 2 DPE when inoculated pigs had on average 1.3 log_10_ eq. CFU/sample higher loads than contact-infected pigs. In the dual-group the inoculated pigs showed 1.8 and 2.2 log_10_ eq. CFU/sample higher mean serotype 2 loads than their contact pigs on DPE 1 and 2, respectively. Mean serotype 9 loads did not differ between inoculated and contact pigs in any of the groups.

**Figure 2:**
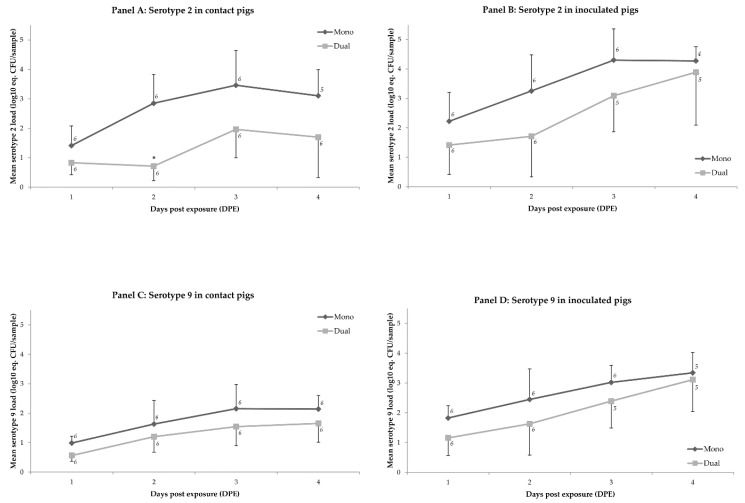
Mean load of *S. suis* serotype 2 (panels A and B) or serotype 9 (panels C and D) in nasal samples (log_10_ eq. CFU/sample) per day post exposure (DPE) visualized for animal categories that differ in type of exposure (i.e., mono or dual), and in mode of infection (i.e., contact or inoculated). Significant differences in levels between mono- and dual-exposed pigs with the same infection mode are marked with (*) if *p* < 0.05 or (**) if *p* < 0.01. The bar at each data point reflects the standard deviation. Number of observations per data point is shown close to that point.

In nasal samples, mean serotype 9 loads did not differ between the mono- and dual-group on any of the days (*p* > 0.2) (Table 3). The mean nasal loads of serotype 2 did not differ between the mono- and dual-group, except at 2 DPE when there was a reduction of on average 2.2 log_10_ eq. CFU/sample (*p* = 0.01) in the contact pigs of the dual-group compared to the contact pigs of the mono-group (Figure 2; Table 4).

### 2.2. Transmission

All contact pigs in the mono-groups were colonized within one day after pairing with the inoculated pigs, and this applied to both serotypes. Within two days, all contact pigs in the dual-group were colonized with both types. For serotype 2, the transmission rates were estimated at 29.4/day (95% C.I.: 0–∞) in the mono-group, and 2.9/day (95% C.I.: 1.2–6.9) in the dual-group (*p* = 0.99). The transmission rates for serotype 9 were 67/day (95% C.I.: 0–∞) and 4.1/day (95% C.I.: 1.6–10.6), for the mono- and dual-group, respectively (*p* = 0.99).

### 2.3. Clinical Disease and Mortality 

In all groups fever, lameness, and neurologic signs were observed. Fever was observed in all pigs exposed to serotype 2 only, in seven out of 12 exposed to serotype 9 only (six inoculated and one contact pig(s)), and in all except one contact pig of the dual-group. Lameness was observed in 10 pigs exposed to serotype 2 only (five inoculated, five contact), and the same was seen in the dual-group. In the group exposed to serotype 9 only, six inoculated pigs showed signs of lameness. Neurologic signs were seen in 10 pigs exposed to serotype 2 only (six inoculated, four contact), in five pigs exposed to serotype 9 only (four inoculated, one contact), and in seven pigs of the dual-group (four inoculated, three contact). High fever and sometimes neurologic signs were accompanied by vomiting in four pigs (two inoculated, two contact) of the group exposed to serotype 2 only, and in three pigs of the dual-group (two inoculated, one contact).

**Figure 3:**
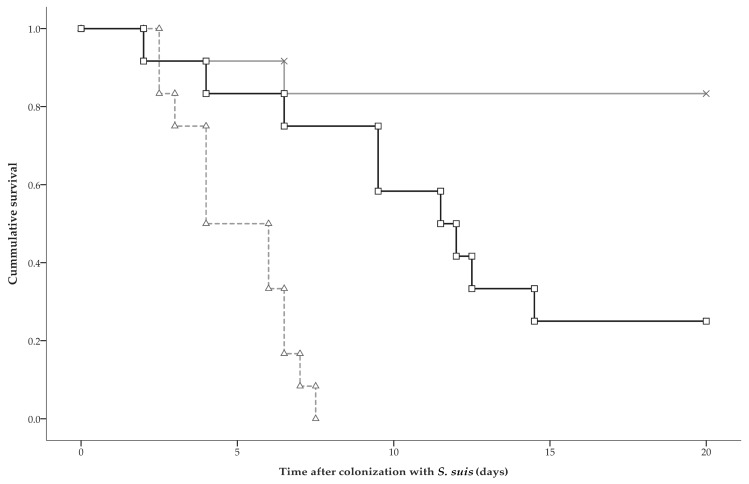
Kaplan-Meier survival curves for groups that were exposed to serotype 2 only (marked by Δ), to serotype 9 only (marked by x), or to both serotypes (marked by □). Survival analysis showed that the hazard for mortality in pigs exposed to both serotypes was 6.59 times (95% C.I.: 1.41–30.86) increased compared to exposure to serotype 9 only, and 6.31 times (95% C.I.: 2.01–19.77) reduced compared to exposure to serotype 2 only. The hazard ratios did not differ significantly between inoculated and contact pigs.

Twenty-three pigs died before the end of the experiment. Mortality occurred in all pigs exposed to serotype 2 only, in two exposed to serotype 9 only (one inoculated, one contact), and in nine pigs of the dual-group (five inoculated, four contact). Mortality occurred between 2–15 days after colonization in inoculated pigs, and between 3–13 days after colonization in contact pigs. Dual exposure reduced the hazard for mortality 6.3 times (95% C.I.: 2.0–19.8) when compared to exposure to serotype 2 only, and increased mortality hazard 6.6 times (95% C.I.: 1.4–30.9) when compared to exposure to serotype 9 only. The hazard for mortality was 41.6 times (95% C.I.: 7.3–236.4) higher after exposure to serotype 2 only compared to exposure to serotype 9 only (Figure 3). The hazard ratios did not differ significantly between inoculated and contact pigs.

In all fatal cases in the group exposed to serotype 2 only as well as in the dual-group, serotype 2 was isolated from internal organ samples of the pigs. In pigs from the group exposed to serotype 9 only, serotype 9 was isolated from internal organs.

## 3. Discussion

The objective of this study was to examine whether inoculation of pigs with *S. suis* serotypes 2 and 9 simultaneously would affect the bacterial load in the oropharyngeal and nasal mucosa, the transmission of either serotype, and the mortality rate of colonized pigs. No significant differences were found in bacterial load or transmission rate of serotype 2 between pigs inoculated with serotype 2 only, or with both types. For serotype 9, a comparable result was found, except for the load on the first day after inoculation, which was approximately 40-fold lower in the pigs inoculated with both types, than in pigs inoculated with one type. In contact pigs exposed to both types, the serotype 2 load was 25-fold to 60-fold reduced for 5 days after colonization in comparison to pigs exposed to serotype 2 only; for serotype 9 a 40-fold reduction in load in contact pigs of the dual-group in comparison to the mono-group was observed on the first day after exposure only. The transmission rates of both serotypes did not differ significantly, neither in the mono-group nor in the dual-group. Finally, simultaneous exposure to both serotypes reduced the mortality of contact pigs due to serotype 2 compared to mortality in contact pigs exposed to serotype 2 only. These findings suggest that ‘natural’ contact exposure to the two serotypes simultaneously affects the clinical outcome of an infection of one of these serotypes (i.e., serotype 2) in a population, possibly by affecting the mucosal load. The relative transmission rates were not affected in our study, suggesting that exposure history, i.e., the consecution at which strains colonize a pig, does not seem to be relevant.

In the pigs exposed to both types, serotype 2 load was more reduced compared to the pigs exposed to one type only, than serotype 9 was. These results suggest competitive interaction between the serotypes, directly (e.g., competition for space, nutrients) and/or indirectly (by e.g., interference of host factors and/or other locally present microbes), on adhesion, replication, and/or clearance of the bacteria. In an in vitro study with these serotypes, it was shown that serotype 9 was more resistant to killing/clearance by animal lysozyme than serotype 2 [30]. Other in vitro studies demonstrated that the biofilm-forming potential of serotype 9 was stronger than serotype 2 strains [31]. Biofilms enable evasion of host immune responses and more resistance against antimicrobials, facilitating persistence and dissemination of the bacteria [32]. Recently, Chuzeville et al. [33] studied in an in vitro setting with isogenic deficient mutants of *S. suis,* the effects of a bacterial protein known as antigen I/II (AgI/II), that was earlier linked to persistence of other streptococci in oral cavity [34], on processes linked to mucosal colonization ability/capacity. It was shown that AgI/II participated significantly in the aggregation of *S. suis* to a salivary glycoprotein, and contributed to biofilm formation, resistance to low pH, as well as bacterial adhesion to extracellular matrix proteins and tracheal epithelial cells. Remarkably, these effects were all much stronger for serotype 9 than for serotype 2. For serotype 9, the effect of AgI/II was also tested in an intranasal porcine model, and revealed the induction of higher mucosal loads; serotype 2 was, unfortunately, not included in that experiment [33]. Altogether, all these results obtained from only in vitro studies indicate a selective advantage of serotype 9 over serotype 2 in colonizing e.g., the upper respiratory tract. 

In another study with these serotypes, it was found that cell constituents of serotype 9 induced a significantly higher activation level of the human Toll Like Receptor 2/6 complex than serotype 2, suggesting a stronger stimulation of the innate immune response at the mucosal level (e.g., induction of pro-inflammatory cytokines, recruitment of phagocytic cells [35]). These findings suggest interference of these mechanisms with bacterial growth, but do not explain the asymmetrical interference found in our experiment. Moreover, we did not observe a difference in bacterial load between serotype 2 and 9 in pigs exposed to one serotype only, suggesting that these mechanisms observed in vitro may not dominate or may not occur, in vivo. Whether these or other biological processes, suggested in other studies with *S. suis* [36,37] or other streptococci like *S. pneumoniae* (e.g., [38,39]), may play a role in interaction between serotypes 2 and 9 has not been determined in vivo either. The animal model presented here may offer a tool to study the effect of these processes on colonization and transmission of these *S. suis* serotypes. In addition, our model or its variants might also be of use in studying the interaction on *S. suis* colonization and transmission between other and/or more than two *S. suis* serotypes that often colonize pigs, between *S. suis* and other pig pathogens that seem to interfere with *S. suis* in vitro, like *Actinobacillus pleuropneumoniae* [40,41] or influenza virus [42], or even between different serotypes of human streptococci that are also able to colonize pigs, like *S. pneumoniae* [43]. 

Simultaneous exposure to serotypes 2 and 9 prolonged the survival time of contact pigs, due to a systemic serotype 2 infection in comparison to pigs exposed to serotype 2 only. This was not observed in inoculated pigs. Whether this is caused by the difference in total dose of *S. suis* exposure between mono-exposed (1 × 10^9^ CFU) and dual-exposed (2 × 10^9^ CFU) inoculated pigs, and/or between inoculated and ‘more naturally’ exposed contact pigs, is unknown and could be subject of further study. In contact pigs, the load of serotype 2 in the mono-group was significantly higher than in contacts in the dual-group. These differences were not observed in the inoculated pigs. These findings in more ‘naturally’ exposed pigs suggest a dose-dependent association between bacterial load and severity of clinical signs [44]. Reduction of bacterial load may be a way to reduce clinical problems if elimination of bacteria in a herd is not feasible. An important condition to evaluate such loads is the availability of a validated test to quantify the presence of (a) virulent strain(s); these are, however, not yet available for conventional pigs. 

Due to logistical and financial limits the number of pigs that could be included in the trial was restricted. As using homogenous groups increases the power of the study, we performed the experiment with caesarean derived, colostrum deprived (CDCD) pigs, to ensure the absence of interference due to serotypes or other micro-organisms already present, and passive maternal-specific immunity. With this study design, it is possible to detect differences in transmission if the reproduction ratio (*R*) in one group is larger than one and in the other below one [45]. No differences in transmission were found, and both serotypes spread extensively (*R* > 1). However, we found differences in serotype 2 load between inoculated and contact pigs in the dual-group, suggesting a difference in infectiousness for serotype 2. This difference may result in differences in transmission between the serotypes if longer transmission chains could be observed, e.g., in experiments where ‘naturally’, contact infected pigs have direct (same pen) or indirect (other pen in stable unit; ‘airborne’) contact with non-infected ones [45,46,47]. Moreover, small difference in transmission can also occur in the field, resulting in significant differences in the number and/or extent of outbreaks, as for example shown for pseudorabies virus [48,49]. If this also applies to *S. suis*, this may result in a shift in distribution of serotypes at the farm level. Small differences in spread cannot be found under experimental conditions, mainly because of the large number of animals required and the associated costs. The use of CDCD pigs and the controlled conditions makes extrapolation to the field more difficult. CDCD and conventional pigs differ in, amongst others, the composition of the mucosal flora and in passive immunity against *S. suis*, which may influence the course of disease [50]. Difference in the course of disease between CDCD and more conventional pigs after *S. suis* infection is suggested by the failure to induce clinical disease after intranasal inoculation with serotype 9 in Specific Pathogen Free (SPF) pigs, both with and without prior mucosal exposure to other pathogenic bacteria (*Bordetella bronchiseptica*) or acetic acid, in some previous experimental studies [44,51], but not in studies using (non-pre-treated) CDCD pigs ([47], this study).

Whether or not differences between CDCD and conventional pigs in, for example mucosal flora (e.g., in previous colonization with other *S. suis* strains), and in passive immunity interfere with colonization of a particular *S. suis* serotype is not known, and requires further field studies. Nevertheless, our study indicated that both serotypes differed in virulence, which is in accordance to earlier studies [44,51], and that virulent strains do not necessarily spread more extensively or rapidly than less virulent strains. Moreover, the presence of one serotype seems to affect the characteristics of the other, suggesting that replacement may occur. Whether this occurs in the field, and has contributed to the serotype shifts observed in practice [6], has to be determined.

## 4. Materials and Methods 

### 4.1. Inoculum

*S. suis* serotype 2 strain 10 and *S. suis* serotype 9 strain 7997 were used as inocula (provided by H. Smith, Wageningen Bioveterinary Research (WBVR), Lelystad, the Netherlands). Both strains contain genes for suilysine (SLY) and muramidase-released protein (MRP), and strain 10 also has a gene encoding for extracellular factor (EF). Strain 10 belongs to MultiLocus Sequence Typing (MLST) clonal complex 1 (CC1), and strain 7997 to CC16.

An aliquot was taken from a −80 °C stock and cultured overnight on agar plates at 37 °C and 5% CO_2_. One colony was suspended in 10 mL Todd-Hewitt broth (TH) (BioTrading, Mijdrecht, the Netherlands), and incubated at 37 °C for 3–4 h until an optical density of 0.5–0.6 at 600 nm was reached. This suspension was stored overnight at 4 °C, and was subsequently diluted tenfold in TH and cultured for 2 h at 37 °C until an optical density of 0.5–0.6 at 600 nm. A total of 100 mL suspension was washed twice and suspended in 10 mL (for inoculation with one serotype) or 5 mL (for inoculation with both serotypes) physiologic saline solution. The bacterial concentration of the final suspension was for each strain 2–3 × 10^8^ CFU per mL for the pigs exposed to one serotype (‘mono-group’), and 4–6 × 10^8^ CFU/mL for the pigs exposed to both types (‘dual-group’).

### 4.2. Pigs 

*S. suis*-free Landrace x Yorkshire pigs were used. These pigs (n = 36) were obtained by caesarean section from sows from the farm of the Faculty of Veterinary Medicine, Utrecht, the Netherlands. The pigs were raised without colostrum. They were first fed with milk replacers and after 2–3 weeks of age there was a transitional period to gamma-irradiated pelleted concentrates (Trouw Nutrition, Amersfoort, The Netherlands). Probiotic bacteria *Enterococcus faecium* (M74^®^, Chr. Hansen, Hørsholm, Denmark), *Bacillus licheniformis* (DSM 5749), and *Bacillus subtilis* (DSM 5750) were added to the feed. Until the start of the experiment the pigs were successively housed in groups of 4–5 animals in isolators (weeks 0 to 4) and altogether in one ground floor pen (weeks 5 and 6).

### 4.3. Experimental Design

**Figure 4:**
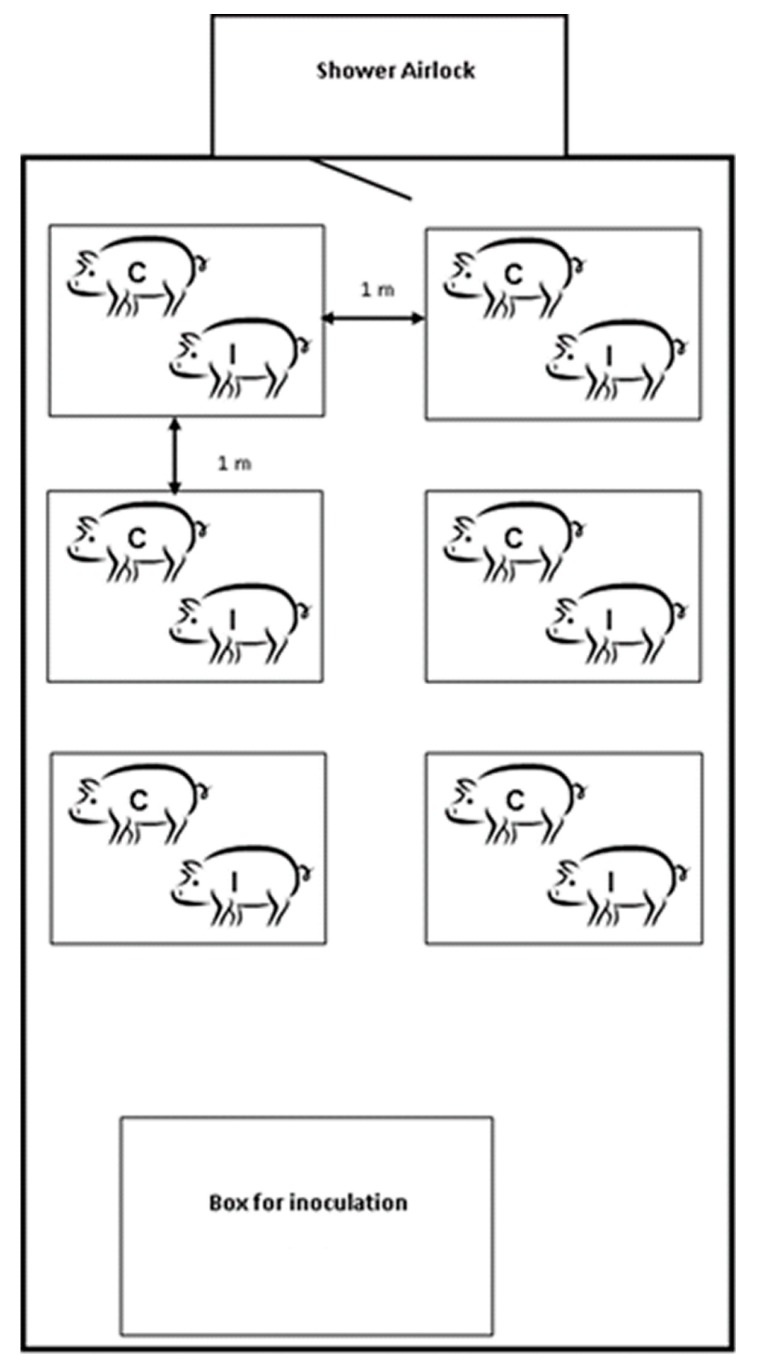
Design of one stable unit as used in the animal experiment. In the unit, each of the six small boxes contained one pig inoculated with *S. suis* (I) and one contact exposed pig (C). The largest box was only used to house the inoculated pigs during inoculation and the following 48 h. In the study, three similar stable units were used. Within a unit, all inoculated pigs were either inoculated with serotype 2 (Group 1), or with serotype 9 (Group 2), or with both serotypes (Group 3).

The experiment was performed in three separated stable units in one building. All stable units had positive air pressure conditions. Each unit had its own ventilation system and hygiene barrier (including shower and airlock). Within each unit, 12 pigs were housed pair-wise in boxes that were separated by a distance of 1.0 m (Figure 4). The boxes had closed walls (height: 80 cm) and a plasticized iron grid floor with rubber lying area, and contained a feeding trough, drinking nipple, and bite sticks. The total ground area was 1.2 m^2^ per box.

Pigs in Unit 1 were exposed to serotype 2 (Group 1), in Unit 2 to serotype 9 (Group 2), and in Unit 3 to both serotypes 2 and 9 (Group 3). To start the infection chain, one pig in each pair was intranasally inoculated with 5 mL saline containing 1 × 10^9^ CFU *S. suis* serotype 2 (Group 1), 1 × 10^9^ CFU *S. suis* serotype 9 (Group 2), or both serotype 2 and 9 in doses of 1 × 10^9^ CFU each (2 × 10^9^ CFU *S. suis* in total) (Group 3) [52]. Inoculation was performed in a ground pen located at a distance of more than 1.5 m from the nearest boxes (Figure 4) and at two days before the entrance of the non-inoculated, contact exposed pigs (contact pigs) in that unit to prevent infection of these contact pigs by the inoculum directly. Two days post inoculation (DPI), the inoculated pigs and the contact pigs were moved to the boxes to form pairs. The pigs were then six weeks old. Pigs were randomly assigned over the experimental groups (Groups 1, 2 and 3), pairs, and modes of infection (inoculated or contact). To prevent infections by animal handling, animal handlers showered and changed clothes before unit entrance, and wore face masks, sterile gloves, and hair covers. Coveralls, gloves, and overshoes were changed before treatment of each box. The experiments were approved by the Animal Care and Ethics Committee of Utrecht University, in accordance with the Dutch law on experimental animals (approval number DEC 2011.II.08.125).

### 4.4. Clinical Inspection

Pigs were inspected daily at 8.00 h and 19.00 h, and clinical observations and rectal temperatures were recorded. Diseased pigs were treated with fentanyl (10 μg/kg body weight), and severely affected pigs were euthanized for ethical reasons. Remaining pigs were sacrificed at the end of the experiment, which was at 21 DPI (similar to 21 DPE for inoculated and 19 DPE for contact pigs).

### 4.5. Sampling

Tonsillar brushing samples were taken in all pigs at similar time points, starting at five days and one day before inoculation. After inoculation for inoculated pigs samples were taken daily at 1–14 and 17 DPE, and at the experiments end (21 DPE). For the contact pigs this was at 1–12, 15 and 19 DPE. Per box, the contact pig was sampled before the inoculated pig. To obtain a tonsillar brushing sample the mouth was opened by a sterile steel wedge, and both palatine tonsillar areas were brushed for 3 s each with a sterile toothbrush. Nasal samples were taken at 1–4 DPE for both inoculated and contact pigs by rotating a swab (type MW102, MedicalWire and Equipment, Corsham, UK) in both nostrils for 2 s each. The brush and swab heads were cut off with sterile wire-cutters, put in separate sterile tubes containing 10 mL (tonsil) or 1.3 mL (nose) saline, and transported to the laboratory immediately. In the lab the tubes were thoroughly mixed, and 1 mL (tonsil) or 0.5 mL (nose) of each sample suspension was transferred into a vial. The vials were stored at −20 °C until further processing. On all pigs post-mortem examination was performed. Macroscopically affected organs were sampled for bacterial examination.

### 4.6. Laboratory Tests

#### 4.6.1. Bacterial Examination

To test the *S. suis* status of the pigs before the experiment, the samples taken before inoculation were submitted to PCR (see below) and to selective bacterial examination (SBE) [52]. For SBE, briefly, 10-fold serial sample dilutions (10^1^ to 10^4^) were made, and 50 μL of the undiluted sample and each dilution was plated on a selective agar plate containing Columbia agar, 6% sheep blood, 0.2 μg/mL crystal violet and colistin/ oxolinic acid (BioTrading) [52,53]. The culture plates were incubated at 37 °C and 5% CO_2_. Colonies that were suspected to be *S. suis* on the basis of colony morphology were subcultured and tested for amylase activity [54]. Isolates that showed amylase activity were further tested with API 20 Strep (bioMérieux, Marcy l’Etoile, France), and serotyped by slide agglutination with *S. suis* serotype specific antisera (serotype 1, 2 and 9) (WBVR) and PCR (see below).

Each sample taken from a lesion seen at necropsy was plated on two Columbia agar plates containing 6% sheep blood and one MacConkey agar plate (Biotrading). All three plates were incubated for 18–24 h at 37 °C; one Columbia agar plate at 5% CO_2_, and the other plates at a normal atmosphere. Colonies that were suspected to be *S. suis* based on colony morphology were selected (in total 10 per animal), subcultured, tested for amylase-activity and serotyped by slide agglutination (see above) and by PCR (see below).

All amylase-positive isolates were suspended in 0.5 mL Tris-HCl EDTA (TE) buffer pH 7.5 (10 mM) and stored at −20 °C until DNA isolation and PCR was performed.

#### 4.6.2. Multiplex Real-Time Quantitative PCR

*S. suis* was detected and quantified using a multiplex qPCR on all nasal and tonsillar samples, as described before [55]. Briefly, after thawing, the samples were mixed and centrifuged (13,000 G, 5 min) in succession, and the supernatant was discarded with a pipette. Subsequently, 200 μL InstaGene™ Matrix (Bio-Rad, Hercules, CA, USA) was added to each sample, as well as 5 μL DNA containing 10 copies of pUC19 as internal control to monitor PCR replication efficiency. DNA was isolated according to the manufacturer instructions of InstaGene™ Matrix (Bio-Rad), with some minor modifications that were recently published [56].

The PCR targeted on the cps9H gene of *S. suis* serotype 9, the cps2J gene of serotype 2 (and 1/2), and on pUC19 (the internal control) by using primers and probes that were described before [52,55,57]. The primers/probe-mix consisting of 300 nM of each cps2J- and cps9H-primer, 125 nM cps9H-probe, 150 nM cps2J-probe, and 100 nM of each pUC19 primer and probe. The PCR mixture (20 μL) contained 10 μL LC480 Probes Master (2×) (Roche Diagnostics, Woerden, the Netherlands), 1 μL of the primers/probe mix, 4 μL PCR-grade water (Roche Diagnostics) and 5 μL template DNA. DNA-amplification was carried out in a LightCycler^®^ 480-II Instrument (Roche Diagnostics GmbH, Mannheim, Germany). The PCR conditions were as follows: 95 °C for 10 min, 45 cycles of 95 °C for 10 s, 58 °C for 30 s, and 72 °C for 30 s, and a final extension at 72 °C. The loads of each serotype in a sample were calculated using standard curves and the LightCycler^®^ 480 algorithm, as described [55]. The minimal detection limits of the qPCR were similar for both serotypes, i.e., 1 eq. CFU per sample in PCR reaction (further designated as ‘eq. CFU/sample’) [55]. Loads higher than the highest concentration in the standard curve (i.e., 1 × 10^4^ eq. CFU/sample) were extrapolated using the algorithm of the LightCycler^®^ 480 Instrument.

Samples were retested if the crossing point of the pUC19 reaction exceeded the previously determined cut-off value of either tonsillar or nasal samples, and excluded from analysis if retesting led to the same result. In our experiment no tonsillar or nasal samples had to be excluded. Before analysis, the loads were log_10_ transformed (log_10_ CFU) for data normalization.

### 4.7. Statistical Analysis

#### 4.7.1. *S. suis* Colonization

Colonization data were used to compare loads of either serotype 2 or 9 after exposure to one serotype, and to evaluate whether a dual infection affects the loads of either of the types. Data of all tonsillar samples were analysed in a linear mixed effects model procedure with the assumption of a normal distribution for the outcome. The load of *S. suis* (log_10_ eq. CFU/sample), either serotype 2 or 9, was used as dependent variable.

Three different models were made; two with data on one serotype (2 or 9) to evaluate the effect of exposure to two types on colonization of a single serotype, and one model with data on both serotypes from the mono-group to compare the colonization capacity between the two serotypes.

The models had a random effect for pig to correct for repeated measurements within the same animal. Time (DPE) and serotype(s) exposure type (mono or dual) were included as explanatory variables. Mode of infection (inoculated or contact) and interactions between the above-mentioned factors were tested by backward selection procedure using the Akaike’s Information Criteria (AIC) to select the best model [58]. The assumptions of normality of residuals and equal variances of the final models were confirmed by visual inspection of quantile-quantile plots, plots of standardized residuals against predicted values, and plots of standardized residuals against all predictor variables. Based on this assumptions check, in the serotype 9 model differences in variances dependent on time (DPE), and for serotype 2 also on mode of infection (inoculated or contact), had to be corrected by adding a variance structure allowing different levels of variances. 

This whole modeling procedure was also performed for comparison of serotype 2 and 9 loads between groups exposed to a single serotype, except that ‘exposure type’ (mono or dual) was replaced by ‘serotype exposure’ (2 or 9) as fixed factor. The same procedure was applied for loads in nasal samples, but no correction for variance structure was needed.

All models were fitted using the statistical program R (version 2.13.0) (R Foundation for Statistical Computing, Vienna, Austria) with use of the LME function from the NLME-library [59].

On the *p*-values of the coefficients’ estimates of the final model, the Benjamini & Yekutieli post-hoc procedure was applied to correct for multiple comparisons. Differences in outcome variables were considered significant if *p* < 0.05.

#### 4.7.2. Animal Survival

Cox proportional hazard (CPH) analysis was performed to evaluate the effect of exposure to both types in comparison to one, and of exposure to serotype 2 only compared to serotype 9 only on animal survival. Time was expressed as day after the first positive nasal and/or tonsillar sample in that pig. In all inoculated pigs this was at 1 DPE, in contact pigs this varied. The CPH model assumes that there is a basal hazard ratio that can be increased or decreased due to factors included in the model. We first added ‘serotype exposure’ (serotype 2 or 9, or both types) and ‘mode of infection’ (inoculated or contact) and their interaction as fixed factors to the model, and frailty to take repeated measurements within one box into account. After backward selection based on AIC values, the interaction was excluded. Compliance with the proportional hazard assumption was tested graphically by plotting the Schoenfeld residuals against time, and by performing a Cox.zph test. The Benjamini & Yekutieli post-hoc procedure was applied to correct for multiple comparisons. Differences in hazard ratios were considered significant if *p* < 0.05.

#### 4.7.3. Transmission

To evaluate the effect of exposure to both *S. suis* serotype 2 and 9 on the spread of each of these types within their host population, and to compare the rate of *S. suis* transmission between hosts exposed to serotype 2 or 9, transmission was quantified per serotype by using a stochastic Susceptible-Infectious (SI) model [60]. With this model, the transmission rate β can be estimated, which is defined as the average number of secondary infections caused by one typical infectious individual in a susceptible population per unit of time [60,61]. For estimation of β we used an approach described previously [52,62], with small modifications. Pigs were classified as being infectious (I) for a particular *S. suis* serotype when tonsillar and/or nose samples were tested positive for that serotype. The effect of exposure to both types, or of exposure to a particular serotype only on transmission parameter β was tested using a generalized linear model with a complementary-Log-Log link function and Log I/N as offset variable [60,62,63]. For evaluation of the effect of exposure to both types, the null hypothesis was that it would have no effect on transmission rate, irrespective of the serotype studied. For comparison of serotypes 2 and 9, the null hypothesis was that both the serotypes would have a similar transmission rate, after simultaneous exposure to both types as well as exposure to one type. The Benjamini & Yekutieli post-hoc procedure was applied to correct for multiple comparisons. Differences were considered significant if *p* < 0.05. Power calculation showed that to detect a difference in transmission between reproduction ratio *R* > 10, as estimated before for *S. suis* serotype 9 [52], and a situation where spread is blocked by intervention (*R* < 1), at least 60 pigs were needed in this experimental set up [45,61]. For logistic and methodological reasons we decided to perform the study in two separate experimental rounds. However, based on the unexpected high disease severity and considering the transmission results in the first round (i.e., absence of transmission reduction; homogeneity in pairs) we decided to cancel a second trial for reasons of animal welfare. Statistical analysis of both animal survival and transmission was performed in R (version 2.13.0).

## Figures and Tables

**Table 1:** Estimated differences in mean tonsillar serotype 2 loads (in log_10_ eq. CFU/sample) between pigs exposed to serotype 2 only (‘Mono’) and to both serotype 2 and 9 (‘Dual’) on different days post exposure ^1^.

	Inoculated Pigs		Contact Exposed Pigs	
Time (DPE) ^1^	Mean load ‘Mono’ Minus Mean Load ‘Dual’ (log_10_ eq. CFU/sample) ^2,3^	*p*-Value	Mean load ‘Mono’ Minus Mean Load ‘Dual’ (log_10_ eq. CFU/sample) ^2,3^	*p*-Value
1	0.64	0.352	1.57	<0.001
2	0.46	0.873	1.40	0.004
3	0.79	0.158	1.72	<0.001
4	0.82	0.158	1.76	<0.001
5	−0.06	0.999	0.87	0.351
6	0.55	0.999	1.48	0.012
7	0.31	0.999	1.24	0.078
8	−0.64	0.999	0.29	0.999

^1^ Data were analysed with a linear mixed regression model. In the final model time (expressed as ‘day post exposure’; DPE), serotype exposure (mono or dual) and mode of infection (inoculated or contact exposed) were included as fixed effects, and pig as random effects; ^2^ The difference in loads is a reflection of coefficient estimate β of the final model. If significant, this difference and its *p*-value are underlined; ^3^ The estimated mean load at 1 DPE in dual group (i.e., the intercept of the final model) was 2.65 log_10_ eq. CFU/sample and 0.80 log_10_ eq. CFU/sample for the inoculated and contact pigs, respectively.

**Table 2:** Estimated differences in mean tonsillar serotype 9 loads (in log_10_ eq. CFU/sample) between pigs exposed to serotype 9 only (‘Mono’) and to both serotype 2 and 9 (‘Dual’) on different days post exposure ^1^.

	Inoculated Pigs		Contact Exposed Pigs	
Time (DPE) ^1^	Mean load ‘Mono’ Minus Mean Load ‘Dual’ (log_10_ eq. CFU/sample) ^2,3^	*p*-Value	Mean load ‘Mono’ Minus Mean Load ‘Dual’ (log_10_ eq. CFU/sample) ^2,3^	*p*-Value
1	1.61	0.004	1.72	<0.001
2	0.86	0.361	0.96	0.240
3	0.79	0.361	0.90	0.227
4	0.29	0.999	0.40	0.999
5	0.00	0.999	0.00	0.999
6	0.00	0.999	0.09	0.999
7	0.13	0.999	0.24	0.999
8	0.03	0.999	0.13	0.999

^1,2^ See legend Table 1; ^3^ The estimated mean load at 1 DPE in dual group (i.e., the intercept of the final model) was 1.46 log_10_ eq. CFU/sample and 1.32 log_10_ eq. CFU/sample for the inoculated and contact pigs, respectively.

**Table 3:** Estimated differences in mean nasal serotype 9 loads (in log_10_ eq. CFU/sample) between pigs exposed to serotype 9 only (‘Mono’) and to both serotype 2 and 9 (‘Dual’) on different days post exposure ^1^.

	Inoculated Pigs		Contact Exposed Pigs	
Time (DPE) ^1^	Mean load ‘Mono’ Minus Mean Load ‘Dual’ (log_10_ eq. CFU/sample) ^2,3^	*p*-Value	Mean load ‘Mono’ Minus Mean Load ‘Dual’ (log_10_ eq. CFU/sample) ^2,3^	*p*-Value
1	0.94	0.233	0.67	0.709
2	0.98	0.203	0.71	0.620
3	0.72	0.650	0.45	0.999
4	0.48	0.999	0.21	0.999

^1,2^ See legend Table 1; ^3^ The estimated mean load at 1 DPE in dual group (i.e., the intercept of the final model) was 0.92 log_10_ eq. CFU/sample and 0.28 log_10_ eq. CFU/sample for the inoculated and contact pigs, respectively.

**Table 4:** Estimated differences in mean nasal serotype 2 loads (in log_10_ eq. CFU/sample) between pigs exposed to serotype 2 only (‘Mono’) and to both serotype 2 and 9 (‘Dual’) on different days post exposure ^1^.

	Inoculated Pigs		Contact Exposed Pigs	
Time (DPE) ^1^	Mean load ‘Mono’ Minus Mean Load ‘Dual’ (log_10_ eq. CFU/sample) ^2,3^	*p*-Value	Mean load ‘Mono’ Minus Mean Load ‘Dual’ (log_10_ eq. CFU/sample) ^2,3^	*p*-Value
1	0.73	0.999	1.29	0.352
2	1.63	0.157	2.19	0.013
3	1.15	0.566	1.70	0.133
4	1.06	0.747	1.61	0.170

^1,2^ See legend Table 1; ^3^ The estimated mean load at 1 DPE in dual group (i.e., the intercept of the final model) was 1.26 log_10_ eq. CFU/sample and 0.34 log_10_ eq. CFU/sample for the inoculated and contact pigs, respectively.
